# Antiplatelet and Anticoagulant Effects of Diterpenes Isolated from the Marine Alga, *Dictyota menstrualis*

**DOI:** 10.3390/md12052471

**Published:** 2014-04-30

**Authors:** Laura de Andrade Moura, Ana Carolina Marqui de Almeida, Thaisa Francielle Souza Domingos, Fredy Ortiz-Ramirez, Diana Negrão Cavalcanti, Valéria Laneuville Teixeira, André Lopes Fuly

**Affiliations:** 1 Departamento de Biologia Molecular e Celular, Instituto de Biologia, Universidade Federal Fluminense, Outeiro de São João Batista, s/n, Centro, Niterói, 24020-141, RJ, Brazil; E-Mails: lauravivimel@gmail.com (L.A.M.); anakarol.marqui@gmail.com (A.C.M.A.); thaisadomingos@yahoo.com.br (T.F.S.D.); 2 Departamento de Biologia Marinha, Instituto de Biologia, Universidade Federal Fluminense, Outeiro de São João Batista, s/n, Centro, Niterói, 24001-970, RJ, Brazil; E-Mails: faortizr@gmail.com (F.O.-R.); dn.cavalcanti@gmail.com (D.N.C.); valerialaneuville@gmail.com (V.L.T.)

**Keywords:** anticoagulant effect, antiplatelet activity, brown algae, *Dictyota menstrualis*, diterpenes

## Abstract

Cardiovascular diseases represent a major cause of disability and death worldwide. Therapeutics are available, but they often have unsatisfactory results and may produce side effects. Alternative treatments based on the use of natural products have been extensively investigated, because of their low toxicity and side effects. Marine organisms are prime candidates for such products, as they are sources of numerous and complex substances with ecological and pharmacological effects. In this work, we investigated, through *in vitro* experiments, the effects of three diterpenes (pachydictyol A, isopachydictyol A and dichotomanol) from the Brazilian marine alga, *Dictyota menstrualis*, on platelet aggregation and plasma coagulation. Results showed that dichotomanol inhibited ADP- or collagen-induced aggregation of platelet-rich plasma (PRP), but failed to inhibit washed platelets (WP). In contrast, pachydictyol A and isopachydictyol A failed to inhibit the aggregation of PRP, but inhibited WP aggregation induced by collagen or thrombin. These diterpenes also inhibited coagulation analyzed by the prothrombin time and activated partial thromboplastin time and on commercial fibrinogen. Moreover, diterpenes inhibited the catalytic activity of thrombin. Theoretical studies using the Osiris Property Explorer software showed that diterpenes have low theoretical toxicity profiles and a drug-score similar to commercial anticoagulant drugs. In conclusion, these diterpenes are promising candidates for use in anticoagulant therapy, and this study also highlights the biotechnological potential of oceans and the importance of bioprospecting to develop medicines.

## 1. Introduction

Nowadays, cardiovascular diseases (thrombosis, venous thromboembolism, stroke and pulmonary embolism) represent the leading cause of disability and mortality worldwide. Such pathologies may occur due to dysfunctions in the hemostatic system, involving the platelets and blood coagulation components [1]. The hemostasis system consists of a complex process to maintain blood flow, but also causes bleeding to stop. Hemostasis is divided into primary and secondary hemostasis, where a platelet plug formation (primary hemostasis) occurs, and the secondary one is the coagulation system, which is composed of a series of enzymes and co-factors (Ca^+2^, platelet phospholipids, vitamin K) to activate thrombin to form a stable fibrin clot. Moreover, thrombin also has a positive feedback function, promoting the activation of specific coagulation factors (factor XI and co-factors V and VIII), stabilizing the fibrin clot, and it also induces platelet aggregation, thereby influencing the formation of plug formation. After a vessel injury, hemostasis has three steps: (1) vasoconstriction; (2) temporary blockage by the platelet plug; and (3) blood coagulation by the forming of a clot that seals the hole until the tissues are repaired. Traditionally, the coagulation cascade is divided into intrinsic and extrinsic coagulation pathways; but nowadays, this model is only useful for *in vitro* diagnostic purposes [2].

The current antithrombotic therapies include vitamin K antagonists, direct thrombin inhibitors, pentasaccharide oral anticoagulants and/or antiplatelet drugs [3]. These therapies have some benefits, but they also have limitations, such as narrow therapeutic windows and indices, resulting in dietary or drug interactions, so that they require monitoring and may produce serious side effects, including gastric disorders, bleeding and thrombocytopenia [4]. Heparin and its analogues are also included among such drugs associated with medication risks [5]. Therefore, alternative antithrombotic therapies are under extensive investigation, and many substances from natural sources are being isolated and studied to counteract these side effects [6].

Marine organisms produce numerous and chemically complex products, identified as secondary metabolites, which display ecological functions, such as defense against herbivores and predators, prevention of biofouling and mediation of symbiosis and reproduction [7]. Apart from these ecological actions, some molecules also exhibit a range of pharmacological effects [8,9], including anti-inflammatory [10], antiviral [11], antiophidic [12], antilonomic [13] or anticoagulant properties [14]. In addition, other natural products have been isolated with antithrombotic activities, such as sulfated galactans from the red alga, *Botryocladia occidentalis* [15], heterofucans from the brown alga, *Canistrocarpus cervicornis* [16], a triterpene saponin from the fruits of *Ilex paraguariensis* [17] and a peptide from the Australian sponge, *Lamellodysidea chlorea* [18]. However, the inhibitory mechanisms of these molecules are still under investigation, with some speculation that they bind directly to thrombin, factor Xa, antithrombin and/or a heparin cofactor II [19].

**Figure 1:**
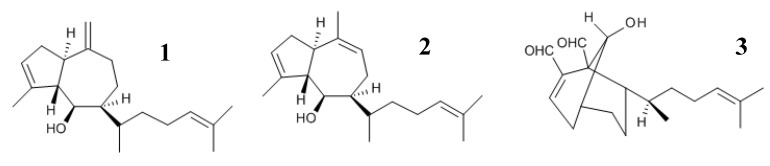
The chemical structure of the diterpenes. **1**, **2** and **3** represent the structure of pachydictyol A, isopachydictyol A and dichotomanol, respectively.

It has already been shown that crude extracts of the marine brown alga, *Dictyota menstrualis*, collected in different areas of the Brazilian coast and prepared in different polarity solvents, exhibited antiplatelet and anticoagulant properties [20,21]. Previous results have shown that the diterpenes, (6*R*)-6-hydroxydichotoma-3,14-diene-1,17-dial, called pachydictyol A (**1**), and its acetate derivative, isopachydictyol A (**2**), as well as 6-Hydroxy-dichotomano-3,14-dieno-1,17-dial (called dichotomanol) (**3**), isolated form *D. menstrualis*, inhibit the human immunodeficiency virus type-1 (HIV-1) replication *in vitro* [22,23]. Moreover, their mechanisms of action and toxicity have been studied, and the results showed that such diterpenes do not induce any cytotoxicity or lethality in mice. Now, the objective of the present study was to evaluate the effects of the diterpenes, pachydictyol A (**1**), isopachydictyol A (**2**) and dichotomanol (**3**) (Figure 1), on platelet aggregation and plasma coagulation.

## 2. Results and Discussion

Hemostasis is a physiologically dynamic process that involves platelet aggregation and blood coagulation and has a major function in the formation of a thrombus, as well as in the prevention of hemorrhage in the case of a vessel injury [24]. The coagulation system is divided into three phases, called initiation, amplification and propagation, which lead to an activation of thrombin, which is a pivotal enzyme generating a fibrin net and activating platelet aggregation. Platelets are equally important to the formation of the thrombus, and after their activation by an agonist (such as collagen, ADP and thrombin), they contribute to the amplification of the blood coagulation system [25,26]. Once uncontrolled, thrombus generation may lead to vascular disturbances and death. Blood disorders represent a global public health problem, and there is not yet a drug sufficiently active, efficient and safe for managing thrombotic disorders [1]. As a result of these problems, new, safer and more effective antithrombotic molecules need to be discovered or designed, but without any, or at least low, side effects.

### 2.1. Effect of Diterpenes on Platelet Aggregation

**Figure 2:**
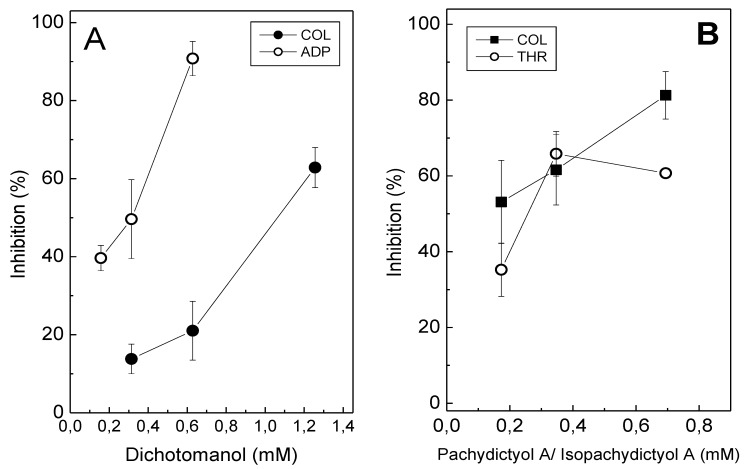
The effect of diterpenes on platelet aggregation. (**A**) Platelet-rich plasma (PRP) was incubated with different concentrations of dichotomanol for 2 min, while stirring, at 37 °C. Then, 16 μg/mL collagen (●) or 15 μM ADP (○) were added to induce platelet aggregation. (**B**) Different concentrations of pachydictyol A/isopachydictyol A were incubated with washed platelets (WP) for 2 min, while stirring, at 37 °C, and then 16 μg/mL collagen (■) or 10 nM thrombin (○) were added to induce aggregation. For both panels, one hundred percent of the platelet aggregation was obtained with supramaximal (able to give a platelet aggregation 70%–80%) concentrations of the agonists in the presence of dimethylsulfoxide (DMSO) after 6 min of reaction. Data are expressed as the means ± SEM of two individual experiments (*n* = 3).

Dichotomanol (0.18 mM–1.38 mM) inhibited platelet aggregation in platelet-rich plasma (PRP) induced by ADP (15 μM) or collagen (16 μg/mL), in a concentration-dependent manner, with IC_50_ values of 0.31 mM and 1.06 mM, respectively (Figure 2A). In contrast, at the highest tested concentration of pachydictyol A/isopachydictyol A (1.38 mM), inhibitions of only 15% and 20% were achieved for collagen- and ADP-induced aggregation in PRP, respectively [27]. Therefore, IC_50_ values could not be determined for the pachydictyol A/isopachydictyol A with PRP. However, when tested on washed platelets (WP), pachydictyol A/isopachydictyol A (0.18 mM–0.7 mM) inhibited aggregation induced by collagen (IC_50_ 0.12 mM) or thrombin (IC_50_ 0.25 mM) (Figure 2B); while dichotomanol (0.32 mM) inhibited only 15% and 30% aggregation induced by collagen or thrombin, respectively [27]. Thus, IC_50_ could not be achieved for dichotomanol on WP. Thrombin was only tested on WP, because it also triggers plasma coagulation and aggregation cannot be monitored. In this way, PRP experiments cannot be performed using thrombin.

ADP, collagen and thrombin bind to different receptors located at the platelet membrane and trigger platelet aggregation through different intracellular pathways [28]. Thus, diterpenes may differentially interact with these receptors, resulting in varying inhibitory profiles in platelet aggregation induced by each agonist. Other factors in the plasma, such as fibrinogen and the Von Willebrand factor, which also participate in platelet aggregation [29], may also have interfered with the inhibitory effect of the diterpenes upon aggregation either with PRP or WP. When testing an effect of any molecules on aggregation, it is mandatory to test them on PRP and WP in order to evaluate if any component of plasma (proteins, lipids, sugars, metals) will interfere on the effect of such molecules, as well as making possible the investigation of a mechanism of action of a molecule on platelets. Pachydictyol A/isopachydictyol A or dichotomanol did not induce the aggregation of platelets, even when tested at the highest concentration (1.38 mM, [27]).

**Figure 3:**
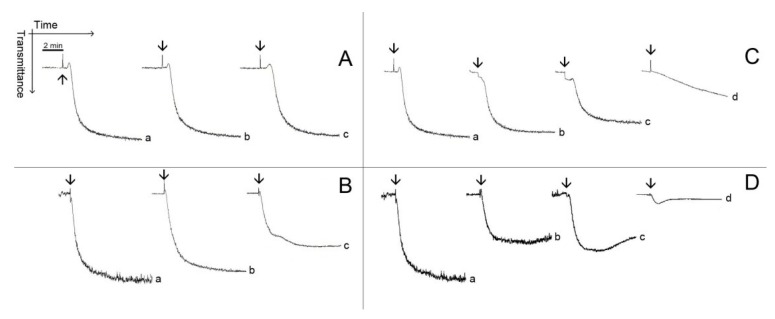
Typical patterns of platelet aggregation with PRP. (**A**,**B**) PRP was incubated for 2 min at 37 °C while stirring with 0.69 mM (Line b) or with 1.38 mM (Line c) of pachydictyol A/isopachydictyol A, then 16 μg/mL of collagen (upper panel) or 15 μM of ADP (lower panel) were added to induce aggregation. (**C**,**D**) PRP was incubated as above, with 0.31 mM (Line b), 0.62 mM (Line c) or with 1.25 mM (Line d) of dichotomanol, and then collagen (upper panel) or ADP (lower panel) was added to the medium. For all panels, Lines a represent PRP incubated with 1% DMSO (v/v, final concentration). The arrows mark the addition of agonists.

**Figure 4:**
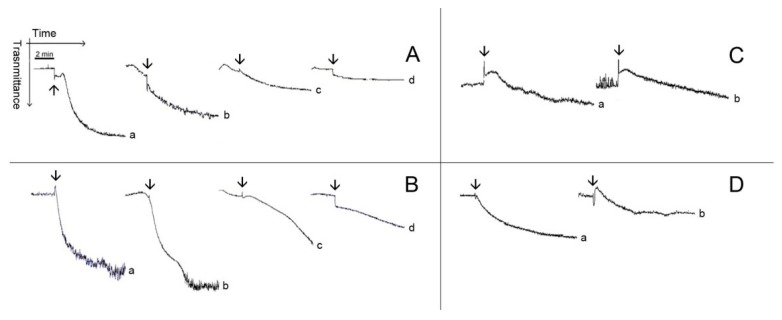
Typical patterns of aggregation with washed platelets. (**A**,**B**) Washed platelets (WP) were incubated for 2 min at 37 °C while stirring with 0.173 mM (Line b), 0.347 (Line c) and 0.694 (Line d) of Pachydictyol A/isopachydictyol A, then 16 μg/mL collagen (upper panel) or 10 nM thrombin (lower panel) was added to induce aggregation. (**C**,**D**) WP were incubated as above with dichotomanol (0.34 mM), and then collagen (upper panel) or thrombin (lower panel) was added to the medium. For all panels, Lines a represent WP incubated with 1% DMSO (v/v, final concentration). The arrows mark the addition of agonists.

Figure 3 and Figure 4 show typical patterns of platelet aggregation on PRP (Figure 3) or WP (Figure 4) in the presence of increasing concentrations of diterpenes. Although diterpenes have inhibited aggregation, shape-change reactions (indicated by the initial decrease in light transmission) were not prevented (Figure 3 and Figure 4). Shape-change is not the second wave of aggregation, as well as it is not a prerequisite for platelet aggregation. Platelet aggregation occurs independently of shape-change, and that shape change is not necessarily followed by aggregation. Some authors hypothesize that the initial changes in light transmission could be due to the microaggregation of the platelets.

### 2.2. Effect of Diterpenes on Coagulation

None of diterpenes exerted a pro-coagulant activity on plasma or on commercial fibrinogen, but they did inhibit coagulation. The pachydictyol A/isopachydictyol A did not prevent coagulation in the prothrombin time test (PT) and moderately delayed coagulation in the activated partial thromboplastin time test (aPTT); while dichotomanol moderately delayed coagulation in the PT and more significantly in the aPTT test (Table 1). All the diterpenes inhibited the coagulation of fibrinogen (FC) induced by thrombin (Table 1).

**Table 1:** The effect of diterpenes on coagulation time analyzed by different methods. For prothrombin time (PT), diterpenes were incubated with plasma for 10 min at 37 °C, and then thromboplastin was added to induce coagulation. For the activated partial thromboplastin time (aPTT), diterpenes were incubated with plasma plus cephalin for 10 min at 37 °C, and then, CaCl_2_ (8.3 mM) was added to induce coagulation. For fibrinogen coagulation (FC), diterpenes were incubated for 10 min at 37 °C with fibrinogen (2 mg/mL), and then, thrombin (10 nM) was added to induce coagulation. Results are expressed as the means ± SEM of two individual experiments (*n* = 8). PAC/ISO, pachydictyol A/isopachydictyol A; DIC, dichotomanol. * *p* < 0.05 when compared with NaCl or DMSO.

Samples	Concentration	Coagulation time (s)		
		PT	aPTT	FC
NaCl	150 mM	21.6 ± 0.5	62.90 ± 1.3	26.4 ± 0.5
DMSO	1% (v/v)	23.3 ± 0.3	65.75 ± 1.0	34.1 ± 1.4
	2% (v/v)	26.7 ± 0.4	73.6 ± 1.8	40.8 ± 1.3
PAC/ISO	0.7 mM	23.9 ± 1.0	72.8 ± 1.5 *	65.3 ± 3.5 *
	1.4 mM	26.9 ± 0.7	87.6 ± 0.9 *	66.7 ± 0.8 *
DIC	0.7 mM	32.4 ± 1.8 *	101.8 ± 4.9 *	47.3 ± 1.6 *
	1.3 mM	41.0 ± 2.6 *	139.9 ± 5.8 *	86.4 ± 2.6 *

### 2.3. Effect of Diterpenes on Thrombin Activity

**Figure 5:**
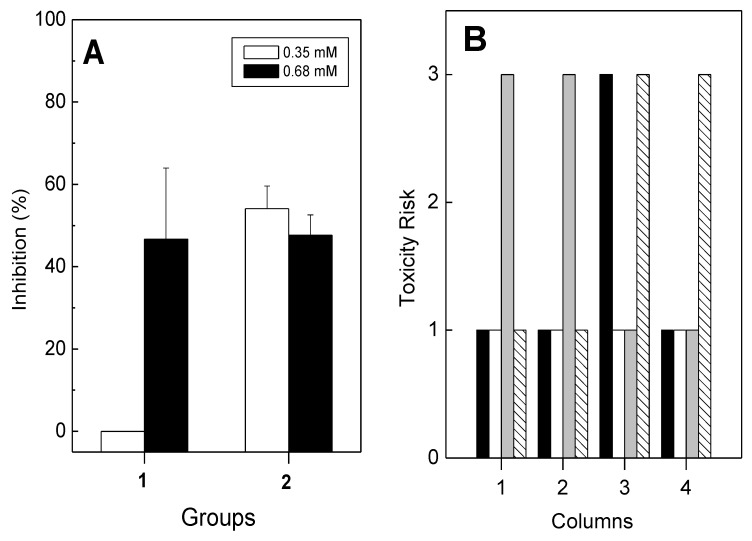
(**A**) Pachydictyol A/isopachydictyol A (Group 1) and dichotomanol (Group 2) were incubated with thrombin (40 nM) for 10 min at 37 °C. After, S-2238 (0.5 mM) was added to the medium, and the reaction was monitored at A 405 nm during 1200 s. One hundred percent of the activity was determined as the difference between values at Absorbance 405 nm obtained at the end and at the beginning of the reaction. Data are expressed as means ± SEM of two individual experiments (*n* = 4); (**B**) The theoretical toxicity risk was evaluated for pachydictyol A/isopachydictyol A (Column 1), for dichotomanol (Column 2), acetyl salicylic acid (Column 3) and warfarin (Column 4). The risks of mutagenicity (black columns), tumorigenicity (white columns), irritability (gray columns) and reproductive negative effects (striped columns) are shown on the Y-axes as numbers and represent one for low, two for medium and three for high.

Pachydictyol A/isopachydictyol A and Dichotomanol alone inhibited platelet aggregation (Figure 2, Figure 3 and Figure 4) and coagulation (Table 1). This inhibitory effect of diterpenes may be due to their ability to inhibit the catalytic activity of thrombin. Therefore, the effect of diterpenes on the enzymatic activity of thrombin was performed using a commercial chromogenic substrate, S-2238, that is specific for the thrombin enzyme and regularly used to evaluate its catalytic activity. As seen in Figure 5A, diterpenes inhibited the enzymatic activity of thrombin, since the hydrolysis of S-2238 by thrombin was diminished. At 0.35 mM, pachydictyol A/isopachydictyol A failed to inhibit hydrolysis, while dichotomanol had 50% inhibition (Figure 5A). However, at higher concentrations (0.68 mM), all diterpenes recorded over 50% inhibition.

The anticoagulant effect of diterpenes suggests that pachydictyol A/isopachydictyol A interferes only in the amplification pathway of the coagulation system, while dichotomanol acts in both, the initiation and the augmentation processes. Moreover, the diterpenes also inhibited the enzymatic activity of thrombin that is a serine protease involved in blood coagulation and platelet aggregation [24]. Thrombin is also responsible for converting fibrinogen into insoluble fibrin, as well as activating other blood coagulation factors (factors XI, VIII and V), thus reducing blood loss. Besides acting on hemostasis, thrombin has a pro-inflammatory action, stimulating the proliferation and migration of smooth muscle cells and apoptosis [30,31]. However, all of those effects are not entirely dependent on the active site of thrombin, since two positively charged domains, called Exosites 1 and 2, participate, as well. The former binds to fibrinogen, platelet receptor PAR-1 and factors V and Va, and the latter is referred to as the heparin-binding site [32]. The interaction of some anticoagulant molecules, such as hirudin, with the thrombin exosites does not inhibit its catalytic activity [33]. The pachydictyol A/isopachydictyol A mixture and dichotomanol inhibited coagulation and platelet aggregation induced by thrombin, and it could be speculated that this inhibitory effect may be due to their ability to inhibit the catalytic activity of thrombin, measured by using a specific chromogenic substrate, S-2238, for such an enzyme [34]. In some cases, a substance with high anticoagulant activity, but a low antiplatelet effect, or the opposite, or even a molecule that does not inhibit all the signaling pathway of platelet aggregation or the coagulation cascade, would be interesting, because the hemorrhagic risk can be diminished. Hemorrhage is one of the most dangerous side effects of current antithrombotic drugs [35].

A candidate molecule to be used as a drug should be safe and not have any toxicity to humans. The theoretical toxicity study is an *in silico* evaluation to predict the toxicity risks of molecules based on their chemical structure. Moreover, it describes the disposition of a pharmaceutical compound within an organism. Four criteria are taken into account—absorption, distribution, metabolism and excretion—because they all influence drug levels and the kinetics of the drug exposure to tissues and, hence, the influence of the action or performance of the molecule as a drug. The theoretical toxicity risks of the diterpenes (pachydictyol A/isopachydictyol A (PAC/ISO) and dichotomanol (DIC)) revealed no theoretical toxicity risks for all the parameters (mutagenicity, tumorigenicity and reproductive negative effects) except for a high irritant profile (Figure 5B). Some commercial antithrombotic drugs, such as acetyl salicylic acid (Figure 5B, column 3) and warfarin (Figure 5B, column 4), had high theoretical mutagenic or reproductive effects, but lacked tumorigenic or irritant activity. The drug-score is a multi-parameter theoretical and structure based value, ranging from zero to one, that may be used to judge the compound’s overall potential to qualify as a drug. Moreover, such theoretical approaches are important tools as a first step to drug discovery, because they may reduce the number of test animals required. The drug-score of the diterpenes were also compared with two commercial antithrombotic drugs, acetyl salicylic acid and warfarin, and such theoretical evaluation of diterpenes regarding toxicity and drug-score showed a low toxicity profile and drug-score values similar or better than acetyl salicylic acid and warfarin [27]. In this way, the theoretical results reinforce the potential use of these diterpenes as antithrombotic compounds.

## 3. Experimental Section

### 3.1. Algae Collection and Isolation of Diterpenes

Specimens of *Dictyota menstrualis* (Dictyotaceae, Phaeophyta) were collected by snorkeling during July, 2010, at Praia do Forno, in the city of Armação de Búzios, located to the north of Rio de Janeiro State, Brazil (22°45′42′′ S and 41°52′27′′ W), at depths ranging from 0.3 to 2 m. The algae were washed with local sea water and separated from sediments, epiphytes and other associated organisms. The air-dried algal material (95 g) was extracted in 100% dichloromethane (CH_2_Cl_2_) exhaustively at room temperature, yielding 5 g of CH_2_Cl_2_ crude extract. The mixture of the diterpenes, pachydictyol A (**1**)/isopachydictyol A (**2**) (45 mg) and the 6-hydroxy-dichotomano-3,14-diene-1,17-dial (also called dichotomanol) (**3**) (21 mg) were obtained and identified according to [36], with some modifications. The crude extract of alga (5 g) was subjected to silica gel (0.015–0.045 mm), eluted with CH_2_Cl_2_:AcOEt (8:2), CH_2_Cl_2_:AcOEt (1:1), AcOEt and acetone. The Fractions **2** and **3** (165 mg) containing a mixture (55 mg) of pachydictyol A/isopachydictyol A were further purified by silica gel-column chromatography (eluent: *n*-hexane) obtaining pachydictyol A/isopachydictyol A (45 mg). Fraction **4** (300 mg), enriched in dichotomanol, was subjected to a reverse-phase (C18) chromatography eluted with CH_3_CN, yielding an impure dichotomanol (41 mg), which was further purified by washing with petroleum ether (21 mg). Their structures were analyzed by NMR spectroscopy and are shown in Figure 1. Finally, the diterpenes were dissolved in dimethylsulfoxide (DMSO, 30% v/v) to perform the biological assays.

### 3.2. Platelet Aggregation Assays

The platelet aggregation assays were carried out according to Fuly *et al.* [37], with some modifications, using platelet-rich plasma (PRP) or washed platelets (WP) from healthy volunteer donors. PRP was prepared by the centrifugation of citrated (0.31%, v/v) human whole blood (340 g for 12 min) at room temperature. For WP, blood was collected in EDTA (5 mM) and centrifuged (340 g for 12 min). Then, PRP was further centrifuged at 1300× *g* for 15 min. The pelleted platelets were then resuspended in a calcium-free Tyrode’s solution containing 0.35% w/v BSA and 0.1 mM EGTA (final concentrations), pH 6.5, and washed twice by centrifugation. The final pellet was resuspended in Tyrode-BSA, pH 7.5, without EGTA (TG) and was adjusted to give 3–4 × 10^5^ platelets/μL. Platelet aggregation was measured turbidimetrically (the increasing light transmission of the platelet suspension) using a Whole Blood Lumi-Aggregometer (Chrono-Log Corporation, Havertown, Pennsylvania, USA). Assays were performed at 37 °C in siliconized glass cuvettes using 300 μL of PRP or WP, while stirring, and aggregation was triggered after incubation for 2 min. One hundred percent (100%) of aggregation was taken as the full platelet response obtained with a supramaximal concentration of agonists, ADP, collagen (purchased from Chrono-Log Corporation, Havertown, Pennsylvania, USA) or human alpha-thrombin (from Haematologic Technologies Inc., Vermont, USA), determined 6 min after their addition, and 0% (base line) platelet aggregation was the light transmittance recorded in the presence of PRP or WP alone or after addition of TG or vehicle (1% v/v DMSO). Different concentrations of diterpenes were incubated with PRP or WP for 2 min at 37 °C while stirring, and then, platelet aggregation was triggered by adding agonists, ADP (15 μM), collagen (16 μg/mL) or thrombin (10 nM). Inhibitory effects on platelet aggregation were expressed as the percentual difference in the maximal responses obtained from the platelet in the presence or in the absence of diterpenes, challenged with agonists. The inhibitory concentration (IC_50_) was designed as the concentration of diterpenes able to inhibit 50% of the platelet aggregation. Control experiments were performed in the presence or the absence of DMSO (1% v/v, final concentration).

### 3.3. Coagulation Assays

Prothrombin time (PT) and activated partial thromboplastin time (aPTT) assays were performed according to the manufacturer’s instructions (Wiener laboratories, Rosario, Argentina). For the PT test, diterpenes were incubated with plasma (50 μL) during 10 min at 37 °C, and then, 100 μL of pre-warmed thromboplastin with calcium were added to initiate coagulation. For the aPTT test, diterpenes were incubated with plasma plus 100 μL of the aPTT reagent, cephalin plus kaolin, for 10 min at 37 °C, with a final volume of 200 μL, and the reaction was started by adding CaCl_2_ (8.3 mM, final concentration). For fibrinogen coagulation (FC), diterpenes were incubated for 10 min at 37 °C with commercial fibrinogen (2 mg/mL) in a final volume of 100 μL, and coagulation was then triggered by adding thrombin (10 nM). Coagulation assays were performed on a Multichannel Coagulometer (Amelung, model KC4A, Labcon, Germany), and coagulation time was recorded in seconds. Plasma was obtained from a pool of citrated healthy volunteer donators (diluted with an equal volume of saline) kindly donated from the public blood bank of the University Hospital Antônio Pedro of the Federal Fluminense University. The collection of blood has been approved by the ethics committee of the hospital. Control experiments were performed by mixing DMSO with plasma or fibrinogen, instead of diterpenes.

### 3.4. Hydrolytic Assay

Hydrolysis of the chromogenic substrate, H-D-Phe-pipecolyl-Arg-pNA.2HCl (S-2238), bought from Chromogenix (Milan, Italy), was monitored using a microplate reader (Thermomax, Molecular Devices, Menlo Park, CA, USA) at A405 nm. The diterpenes were incubated with thrombin (40 nM, final concentration) for 10 min at 37 °C, and then, the reaction was triggered by adding S-2238 (0.1 mM, final concentration). The reaction was monitored during 20 min at 37 °C. Control experiments were performed by incubating thrombin with either DMSO (1% v/v) or saline.

### 3.5. Theoretical Toxicity Study

The theoretical studies of toxicity and drug-score were performed using the software, Osiris Property Explorer [38], and four theoretical toxicity risks were calculated: mutagenicity, tumorigenicity, irritability and reproductive negative effects. They were calculated based on the structure of molecules and were compared with known structures obtained from a databank. The theoretical parameter drug-score was calculated, as well. The drug-score is a multi-parameter value that combines drug likeness, molecular weight and toxicity risks in one handy value in order to be used to judge the molecule’s overall potential to qualify as a drug.

### 3.6. Statistical Analysis

The results were expressed as the means ± standard error (SEM) obtained with the number of experiments performed indicated. The statistical significance of differences among experimental groups was evaluated using the Student *t*-test. *p*-values of <0.05 were considered statistically significant.

## 4. Conclusions

Taken together, these algal diterpenes have revealed antithrombotic properties, and the results have shown the value of bioprospecting research for discovering novel products with promising pharmacological effects for drug development. However, it is important to state that the theoretical or *in vivo* toxicity is not fully reliable, nor does it guarantee that diterpenes are completely free of any toxic effect, but rather should be used as a base to guide the selection of compounds for future assays. Natural products, such as diterpenes, could also be used to help to design structure-based anticoagulant or antiplatelet drugs in order to improve antithrombotic therapies, since diterpenes have low or no toxicity and do not promote side effects. Moreover, agents from non-mammalian sources may diminish the risk of contamination with a foreign body or pathogenic substances. On the other hand, commercial therapeutic drugs usually promote side effects, such as hemorrhage, hypotension and thrombocytopenia.
